# Hyaluronic acid as a treatment for refractory *Bacillus* Calmette–Guérin-induced cystitis: A narrative review

**DOI:** 10.14440/bladder.2024.0066

**Published:** 2025-03-24

**Authors:** Ayoub Gomati, Mai Teggaz, Mazen Allam, Wasim Mahmalji

**Affiliations:** Department of Urology, Wye Valley Trust, Hereford County Hospital, Hereford HR1 2EX, United Kingdom

**Keywords:** Hyaluronic acid, *Bacillus* Calmette–Guérin-induced cystitis, Intravesical therapy, Glycosaminoglycan layer, Combination therapy, Chondroitin sulfate, Cystistat

## Abstract

**Background:**

Hyaluronic acid (HA) instillation has emerged as a potential alternative treatment for *Bacillus* Calmette–Guérin (BCG)-induced cystitis, a common complication of BCG intravesical therapy for non-muscle-invasive bladder cancer (NMIBC). BCG-induced cystitis presents with symptoms similar to bacterial infections, such as urinary urgency, frequency, and pain. Conventional treatments, such as BCG discontinuation, antibiotic therapy, and corticosteroid use, are often insufficient. HA therapy works by restoring the bladder’s glycosaminoglycan layer, reducing inflammation, and promoting tissue repair.

**Objectives:**

This narrative review assessed the efficacy and safety of HA in managing BCG-induced cystitis based on a literature search of PubMed, Google Scholar, and Cochrane databases, identifying seven relevant studies.

**Conclusion:**

HA treatment has been associated with improvements in bladder symptoms, including reductions in pain, urgency, and frequency, as well as an increase in bladder capacity. Combination treatments with chondroitin sulfate or pirarubicin demonstrated superior outcomes compared to HA alone. While the studies reported minimal adverse effects, variability in study design, sample sizes, and follow-up durations limited the strength of the evidence. These findings suggest that HA can be safely administered to NMIBC patients alongside BCG therapy with minimal side effects and no adverse impact on treatment outcomes.

## 1. Introduction

Intravesical instillation of *Bacillus* Calmette–Guérin (BCG) has been a cornerstone of immunotherapy for non-muscle-invasive bladder cancer for over four decades, proving to be an effective adjuvant treatment. BCG therapy enhances an immune response known as trained immunity, which plays a vital role in cancer control by inducing a localized immune response. However, despite its therapeutic benefits, BCG treatment is often associated with adverse effects, with BCG-induced cystitis being the most common local complication.

BCG-induced cystitis is a debilitating condition characterized by a range of distressing symptoms, including urinary frequency, urgency, nocturia, hematuria, and moderate-to-severe pelvic pain. In severe cases, the condition may progress to the extent where cystectomy is necessary. This condition afflicts approximately 30 – 60% of patients undergoing BCG therapy. Despite variations in treatment schedules, age, and BCG strains, the incidence of cystitis-related symptoms remains consistent. As outlined in clinical guidelines, current management strategies for BCG-induced cystitis involve a stepwise approach. This includes halting BCG instillations, administering empirical antibiotics, and potentially using anti-tuberculosis medications combined with corticosteroids if symptoms persist. In extreme cases, radical cystectomy may be considered for patients with refractory symptoms or severely contracted bladders.

Given the significant impact of BCG cystitis on patients’ quality of life and the frequent discontinuation of BCG therapy due to these side effects, exploring alternative treatments has become a priority. Hyaluronic acid (HA) instillation has emerged as a promising therapeutic option. HA, a naturally occurring glycosaminoglycan (GAG), aids in restoring the bladder’s mucosal layer, reducing inflammation, and promoting tissue repair. This therapeutic effect of HA has been clearly shown in cases of severe inflammation and scarring in interstitial cystitis (IC).^1^ This systematic review aimed to assess the efficacy and safety of HA in managing BCG-induced cystitis, synthesizing current research to guide future treatment protocols.

## 2. GAG-based therapy for BCG-induced cystitis

The GAG layer functions as a protective mucus barrier over the urothelium, regulating permeability and shielding the bladder wall from irritants.^2^ This layer is primarily composed of HA, chondroitin sulfate (CS), keratin sulfate, and heparin sulfate. When compromised due to conditions such as bladder infections, IC, radiation cystitis,^3^ bladder cancer treatments, or post-intravesical BCG instillations, the epithelium becomes more permeable. This increased permeability allows potassium ions, bacteria, and urinary solutes to penetrate bladder tissues. As a result, an inflammatory response is triggered, leading to symptoms such as urgency, frequency, and pain. These symptoms have been reported in other severe bladder inflammation, such as IC,^1^,^4^ causing significant distress to patients. Replenishing these GAG components offers a promising therapeutic approach by restoring the protective barrier function of the GAG layer.

HA, in particular, helps restore the GAG layer by reducing epithelial permeability through the stimulation of tight junction proteins. It also exhibits anti-inflammatory properties by inhibiting mast cell activation and immune cell infiltration. Studies showed that CS, another key GAG component, plays a crucial role in bladder barrier function, making the combination of HA and CS an effective treatment option.^2^ This combination, which is administered weekly for 8 weeks, has been demonstrated to significantly improve bladder symptoms, particularly in patients with IC.

In the context of BCG-induced cystitis, HA plays a dual role. First, it helps restore the GAG layer to prevent bladder irritation. Second, when combined with paclitaxel in bioconjugates such as paclitaxel-hyaluronan conjugate, HA enhances antitumor activity in BCG-refractory bladder cancer. A recent retrospective series has shown promising results for the paclitaxel-hyaluronan conjugate, demonstrating a high rate of complete response in patients with BCG-refractory carcinoma *in situ*. However, further research is needed to confirm these promising results.^2^

The following are the general observations derived from the studies presented in Table 1 on HA use for BCG-induced cystitis treatment:

**Table 1:** Clinical studies on HA for *Bacillus* Calmette–Guérin-induced cystitis treatment

Study/country	Study design	Participants	Treatment	Results	Critique	Level of evidence
Sommariva *et al*.^5^/Italy	Prospective study	69 patients (24 BCG cystitis)	Weekly instillations of sodium hyaluronate (40 mg/50 mL); 8 – 24 weeks	Bladder capacity increased from 58.4 mL to 283.7 mL (*p*<0.0001), and pain VAS score dropped from 8.6 to 0.9	The lack of a control group and specific cohort may limit the applicability	1b (Prospective study)

Li *et al*.^6^/China	Multicentered, randomized controlled trial	120 patients	Cystistat (HA-based) with THP following TURBT; the control group received only THP	VAS score for pain improved by 92.92% in the HA group (*p*<0.01), and frequency and nocturia improved significantly	There are no significant improvements in hematuria or other bladder irritation symptoms and needs more extensive studies	1b (Randomized controlled trial)

Topazio *et al*.^7^/Italy	Prospective, randomized controlled trial	30 patients	Group B received HA instillations along with BCG; Group A had BCG only	VAS score improved from 5.8 to 4.2 (*p*=0.0001), with improvement in IPSS scores and urinary symptoms, and recurrence in seven patients	Small sample size and short 6-month follow-up	1b (Randomized controlled trial)

Imperatore *et al*.^8^/Italy	Retrospective study	20 patients	8 weekly instillations of combined HA/CS therapy for chemical cystitis	VAS scores improved for urgency and pain, urinary frequency reduced, and sustained improvements after 6 months and 1 year	Small sample size and retrospective design limit generalizability	2b (Retrospective study)

Lee *et al*.^9^/Korea	Retrospective cohort analysis	217 patients	Group A: BCG only, Group B: BCG+oral PPS (100 mg thrice daily during BCG treatment)	Discontinuation rate: 15.6% (Group A) versus 6.3% (Group B) (*p*=0.034), pyuria higher in Group B (*p*=0.029), antibiotic use higher in Group A (*p*=0.001), and no difference in 1-year recurrence rate (*p*=0.507)	Retrospective design and needs further prospective validation	2b (Retrospective cohort study)

Pichler *et al*.^2^	Retrospective case series	Five patients	Weekly instillations of HA+CS for 6 weeks	There was a significant reduction in urinary urgency and bladder pain, QoL improved, and no tumor recurrences were observed	A small sample size (five patients) and longer follow-ups needed to validate the results	Four (Case series)

Abbreviations: BCG: *Bacillus* Calmette–Guérin; CS: Chondroitin sulfate; HA: Hyaluronic acid; IPSS: International prostate symptom control; PPS: Pentosan polysulfate; QoL: Quality of life; THP: Pirarubicin; TURBT: Trans-urethral resection of bladder tumor; VAS: Visual analog scale.

(i) Improvement in symptoms: All studies consistently reported significant improvements in bladder-related symptoms following HA or HA + CS treatments. Symptoms such as bladder pain, urgency, frequency, and incontinence decreased, with studies noting improvement in Visual Analog Scale (VAS) and quality of life scores, such as the International Consultation on Incontinence Questionnaire–Urinary Incontinence Short Form and the Incontinence Quality of Life questionnaires. For example, the study by Imperatore *et al*.^8^ showed a reduction in urgency and pain, with sustained benefits over a 6-month to 1-year follow-up. These findings confirm the therapeutic potential of HA and CS in managing BCG-induced cystitis
(ii) Small cohorts: A limitation in most of the studies is their small sample size. Several studies^2^,^8^ involved small cohorts ranging from 5 to 20 patients. This restricts the statistical power and generalizability of the results, leading to a need for larger-scale trials to validate these findings
(iii) Prospective versus retrospective designs: The studies feature both prospective and retrospective designs, with prospective trials providing more substantial evidence. Retrospective studies^2^ are more prone to bias; however, they still demonstrate symptomatic improvements and oncological safety. The lack of randomized, controlled prospective studies limits the strength of conclusions regarding the efficacy of these treatments
(iv) Combination therapy: Studies that explored the combination of HA with other agents, such as CS or pirarubicin,^6^ showed more robust outcomes than HA monotherapy. For example, combined HA + CS therapy resulted in faster and more durable symptom relief compared to using HA alone, highlighting the potential for combination therapy in treating BCG-induced cystitis.

## 3. Other treatment options for BCG cystitis

A systematic review by Poletajew *et al*.^10^ in 2019 focused on the prevention and treatment options for cystitis in bladder cancer patients undergoing intravesical BCG immunotherapy. The review identified local adverse effects, such as cystitis, as the most common clinical symptom in these patients. Following a comprehensive literature search, relevant studies were analyzed. Key findings include:

(i) Prevention strategies: Reducing the BCG dose was associated with lower local adverse effects in some studies, but there was no difference in others. Intravesical HA instillations and oral antibiotics (e.g., prulifloxacin and ofloxacin) were also identified as potentially effective in preventing cystitis
(ii) Treatment options: To treat BCG-related cystitis, oral pentosan polysulfate and a combination of intravesical HA and CS were highlighted as potential options. However, these were based on limited evidence from small studies
(iii) Challenges: The studies were highly heterogeneous, differing in BCG strains, treatment schedules, and endpoints. In addition, there was no conclusive evidence to support any intervention being both oncologically safe and effective at preventing or treating BCG-induced cystitis
(iv) Summary: Current management of BCG-related cystitis is primarily symptomatic, and more research is needed to identify both effective and oncologically safe treatments.

In summary, while several strategies have shown potential, there is no definitive, well-established approach for preventing or treating BCG-related cystitis. Further large-scale trials are necessary to provide more substantial evidence, as shown in Table 2.^11^

**Table 2:** Management strategies for *Bacillus* Calmette–Guérin cystitis induced lower urinary tract symptoms

Target	Rationale	Drug/Medication	Mechanism	Clinical evidence
Detrusor smooth muscle contraction^12^-^14^	Decreasing detrusor muscle contractions may reduce urinary urgency and frequency. First-line therapies for detrusor overactivity in OAB patients.	Oxybutynin: Antimuscarinic	Induces bladder relaxation through inhibition of muscarinic receptors on the detrusor smooth muscle.	Oxybutynin significantly reduced urinary urgency compared to placebo (*n*=60, *P*<0.001). Oxybutynin significantly increased urinary frequency (*p*<0.01) and burning on urination (*p*<0.05) compared to placebo (*n*=50).

		Mirabegron: b3-adrenoceptor agonist	Increases cyclic adenosine monophosphate concentrations, causing relaxation of bladder smooth muscle.	Mirabegron significantly reduced urinary frequency, urgency, nocturia, and pain when compared to placebo (*n*=160, *P*<0.001).

Inflammation^12^	Acute bladder inflammation has a positive correlation with an increase in urinary urgency, frequency, and pain.	Celecoxib: COX-2 Selective NSAID	Reduces inflammation through COX-2 inhibition.	Celecoxib significantly reduced urinary frequency (*p*<0.001), urinary urgency (*p*<0.001), and dysuria (*p*<0.01) when compared to placebo (*n*=60).

Sensory nerves^12^	Peripheral endings of bladder sensory nerves become hypersensitive during BCG cystitis.	Phenazopyridine: Urinary analgesic	Mechanism of action is still not well understood–proposed to interact directly with sensory nerves through TRPM8.	Phenazopyridine significantly reduced urinary frequency (*p*<0.01), urinary urgency (*p*<0.001), and dysuria (*p*<0.01) when compared to placebo (*n*=60).

Bladder permeability^7^,^9^,^15^	Increased bladder permeability caused by inflammation allows toxic waste products in urine to act on bladder sensory nerves, inducing bladder hyperexcitability and pain.	Pentosan polysulfate sodium	Assists in restoring the barrier function of the urothelium.	Patients receiving PPS were significantly less likely to discontinue BCG installations due to LUTS when compared to placebo (*n*=217, 15.6% vs. 6.3%, *p*<0.05). Post-treatment VAS scores were significantly lower in patients receiving PPS compared to controls (*n*=32, *p*<0.01).

		Hyaluronic acid (HA)	Assists in restoring and replenishing the GAG layer.	Individuals with NMIBC (*n*=15) infused with BCG and HA reported significantly lower VAS for pain compared to those infused with only BCG (*p*<0.05).

Abbreviations: BCG: *Bacillus* Calmette–Guérin; GAG: Glycosaminoglycan; LUTS: Lower urinary tract symptoms; NMIBC: Non-muscle invasive bladder cancer; NSAID: Non-steroidal anti-inflammatory drug; PPS: Pentosan polysulfate sodium; VAS: Visual Analog Scale.

**Figure 1:**
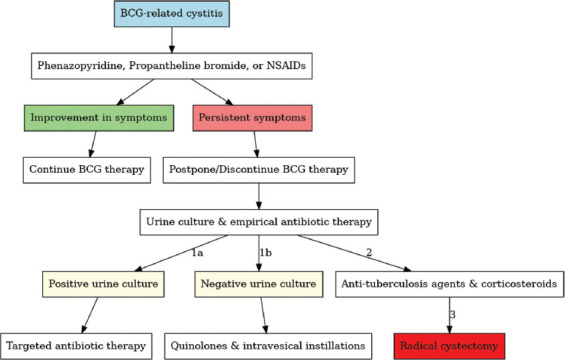
Currently available guidance for the management of Bacillus Calmette–Guérin cystitis

A flowchart based on the European Association of Urology stepwise approach for BCG-related cystitis, as described by Poletajew *et al.*,^10^ outlines a structured management algorithm (Figure 1) of current recommendation.

## 4. Discussion

The limited evidence regarding HA in managing BCG-induced cystitis shows significant promise, particularly in alleviating bladder-related symptoms such as pain, urgency, frequency, and incontinence. Symptomatic relief is reflected in both subjective and objective measures, including reductions in VAS scores and improvements in quality-of-life assessments. However, the reviewed studies are often constrained by small sample sizes, limiting their statistical power and generalizability. This underscores the need for larger, well-designed clinical trials to confirm the long-term benefits of HA treatment.

### 4.1. Impact on clinical decision-making

While evidence supports the use of HA, particularly in combination with CS, clinical decision-making remains hampered by the lack of standardized protocols. Although current studies demonstrated symptomatic relief, urologists require more definitive research to guide long-term management strategies. Incorporating HA as a routine treatment for BCG-induced cystitis in clinical practice could prevent discontinuation of BCG, preserving oncological outcomes while alleviating patient discomfort.

In contrast, a systematic review^10^ of preventive and therapeutic strategies for BCG-related cystitis found no intervention conclusively effective and oncologically safe. HA instillations were highlighted as a promising option, but more extensive, more homogeneous studies are required. Similar to the findings on HA and CS combination therapy, the studies reviewed by Poletajew *et al*.^10^ were limited by small sample sizes, heterogeneous study designs, and short follow-up durations, which create gaps in the evidence base.

In summary, both this review on HA and the broader review of BCG-related cystitis treatments emphasize the need for more robust research. While HA and CS combination therapies show promise, the small sample sizes and varying study designs underscore the need for larger, randomized controlled trials to establish evidence-based guidelines for managing BCG-induced cystitis. At present, management remains largely symptomatic, with no definitive treatment protocol available.

### 4.2. Roles of HA in the treatment of other bladder conditions

HA has garnered considerable attention for its role in treating a variety of bladder conditions beyond BCG-induced cystitis. One of its primary mechanisms is the restoration of the GAG layer of the bladder, which is crucial for maintaining the integrity of the urothelium and acting as a barrier against urinary irritants. When this layer is compromised, the bladder becomes more permeable, leading to inflammation and symptoms such as pain, urgency, and frequency. HA has been used in the treatment of the following conditions:

(i) IC: HA is widely used in the management of IC, also known as bladder pain syndrome, a chronic condition characterized by severe bladder pain, frequent urination, and urgency. In IC, the GAG layer is often damaged, contributing to inflammation and urothelial dysfunction. Intravesical instillations of HA help to replenish the GAG layer, improving bladder wall protection and reducing the entry of irritants that exacerbate symptoms. Studies have shown that HA treatment leads to significant improvements in pain relief, reduced urinary urgency, and overall quality of life in IC patients, mainly when administered in conjunction with other agents such as CS^16^
(ii) Radiation-induced cystitis:^10^ Radiation therapy, commonly used to treat pelvic malignancies, often results in radiation-induced cystitis. This condition causes damage to the bladder mucosa, leading to inflammation, hematuria, and urinary frequency. HA has been used as part of a therapeutic approach to alleviate these symptoms by promoting the healing of the damaged mucosa and restoring the GAG layer. Patients receiving HA instillations for radiation cystitis often report a reduction in bladder irritation, hematuria, and discomfort, making HA a valuable option for those suffering from the side effects of radiation therapy
(iii) Chemical cystitis: Exposure to chemicals, such as those used in chemotherapy or certain irritants found in cleaning products, can lead to chemical cystitis. This condition results in inflammation, irritation, and sometimes ulceration of the bladder lining. HA, often in combination with CS, has been shown to provide therapeutic benefits in chemical cystitis by re-establishing the protective barrier of the bladder, reducing inflammation, and promoting tissue regeneration. The use of HA in these cases is particularly beneficial because it addresses the underlying damage to the bladder’s GAG layer, leading to both symptomatic relief and long-term recovery
(iv) Post-operative cystitis: Some bladder surgeries, such as transurethral resection of bladder tumors, can lead to post-operative cystitis. Post-operative cystitis is characterized by bladder inflammation and irritative urinary symptoms. HA instillations are used postoperatively to aid in the healing process and reduce the risk of infection or persistent inflammation. The anti-inflammatory and tissue-repair properties of HA facilitate faster recovery and reduce post-operative complications.

HA plays a critical role not only in providing symptomatic relief but also in addressing the underlying pathology of various bladder conditions, particularly by restoring the compromised GAG layer. By replenishing the GAG layer, HA reduces inflammation, promotes healing, and improves overall bladder function. Given its broad therapeutic potential, HA has become a cornerstone in managing various bladder disorders, with ongoing research exploring new applications and combinations with other agents to enhance its efficacy.

Conditions such as IC, radiation-induced cystitis, chemical cystitis, post-operative cystitis, bacterial cystitis, and COVID-associated cystitis represent different clinical manifestations of the same inflammatory response to bladder injury. This commonality forms the basis of a novel hypothesis suggesting that HA may be a successful treatment strategy across these varied conditions in previous studies.^1^,^4^

However, the published studies are limited by small patient cohorts. More extensive studies with appropriate control groups are needed to assess the effects of HA more reliably. In addition, more extended follow-up periods are necessary to evaluate the long-term responses to HA treatment.

Furthermore, one significant limitation of the published studies is their predominantly descriptive approach. A more quantitative approach, with objective molecular measurements of inflammatory changes in bladder tissues, would enhance the quality of these studies. Recent work has demonstrated the potential of such molecular assessments in other benign urological conditions.^17^

Moreover, analyzing patients undergoing multiple rounds of HA treatment could provide insights into whether subsequent treatments amplify or diminish initial effects over time.

The most significant limitation of the published studies is the lack of control groups. The ideal control group would consist of patients with the same baseline characteristics who receive instillations of a fluid or serum without HA components, allowing the assessment of both the placebo effect and the therapeutic effect of the procedure. Such an approach would be valuable and could serve as a foundation for future studies in this field.

## 5. Conclusion

HA offers a therapeutic approach for managing BCG-induced cystitis by restoring the bladder’s protective GAG layer, reducing inflammation, and improving bladder function. The clinical evidence reviewed shows significant symptom relief, including reductions in pain, urgency, and frequency, and an increase in bladder capacity. Combination therapies with CS have demonstrated superior efficacy compared to HA monotherapy, offering faster and more sustained symptom relief.

In conclusion, our review highlights that intravesical administration of HA appears to be a safe and effective therapeutic option for patients with BCG-induced cystitis, as supported by the positive outcomes reported in the current evidence base. This approach is backed by robust pathophysiological data that reinforces its potential efficacy. However, more extensive, well-designed, controlled studies are necessary to provide further insights into this emerging treatment modality and to validate HA’s effectiveness in managing severe bladder inflammation associated with BCG therapy, particularly in cases refractory to conventional treatments. Furthermore, future research should also focus on generating quantitative evidence to clarify the cellular-level effects of HA on the bladder wall, thereby elucidating its therapeutic mechanisms.

## Data Availability

All data reviewed in this study are available in their respective published articles.
